# Predators can influence the host‐parasite dynamics of their prey via nonconsumptive effects

**DOI:** 10.1002/ece3.6401

**Published:** 2020-06-12

**Authors:** Nicolette Zukowski, Devin Kirk, Kiran Wadhawan, Dylan Shea, Denon Start, Martin Krkošek

**Affiliations:** ^1^ Department of Ecology and Evolutionary Biology University of Toronto Toronto ON Canada; ^2^ School of Public Health, University of California Berkeley CA USA; ^3^ Department of Biology Stanford University Stanford CA USA; ^4^ Center for Population Biology University of California Davis CA USA

**Keywords:** *Chaoborus*, *Daphnia*, indirect interactions, life history, microsporidian, *Ordospora*, parasitism, predation, sex

## Abstract

Ecological communities are partly structured by indirect interactions, where one species can indirectly affect another by altering its interactions with a third species. In the absence of direct predation, nonconsumptive effects of predators on prey have important implications for subsequent community interactions. To better understand these interactions, we used a *Daphnia*‐parasite‐predator cue system to evaluate if predation risk affects *Daphnia* responses to a parasite. We investigated the effects of predator cues on two aspects of host–parasite interactions (susceptibility to infection and infection intensity), and whether or not these effects differed between sexes. Our results show that changes in response to predator cues caused an increase in the prevalence and intensity of parasite infections in female predator‐exposed *Daphnia*. Importantly, the magnitude of infection risk depended on how long *Daphnia* were exposed to the cues. Additionally, heavily infected *Daphnia* that were constantly exposed to cues produced relatively more offspring. While males were ~5× less likely to become infected compared to females, we were unable to detect effects of predator cues on male *Daphnia*–parasite interactions. In sum, predators, prey, and their parasites can form complex subnetworks in food webs, necessitating a nuanced understanding of how nonconsumptive effects may mediate these interactions.

## Graphical abstract

We used a *Daphnia*‐parasite‐predator cue system to evaluate if predation risk affects *Daphnia* responses to a parasite. We investigated the nonconsumptive effects of predator cues on two aspects of host–parasite interactions (susceptibility to infection and infection intensity), and whether or not these effects differed between sexes. Our results indicate that changes in response to predator cues caused an increase in the prevalence and intensity of parasite infections in female predator‐exposed *Daphnia*; however, the magnitude of infection risk depended on how long *Daphnia* were exposed to the cues.

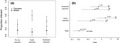

## 1 INTRODUCTION

Organisms do not live in isolation but are instead embedded in tangled webs where they interact with many other organisms. Indeed, species interactions are important determinants of myriad biological patterns. Beyond simple pairwise interactions (e.g., predation or parasitism), multi‐species systems can also be structured by indirect interactions (Bertram, Pinkowski, Hall, Duffy, & Cáceres, 2013; Montoya, Pimm, & Solé, 2006). Indirect interactions are defined by the changing strength of interactions between two species in the presence of a third (Sotomayor & Lortie, 2015). For instance, two species that do not compete for resources may interact indirectly if they share a common predator (i.e., apparent competition) (Hatcher & Dunn, 2011; Holt, 1977). More subtly, indirect interactions between two species can also arise when the phenotype of one species changes in response to the presence of another, and this can occur via a predator influencing its prey without actually predating upon it (nonconsumptive effects) (Abrams, Menge, Mittelbach, Spiller, & Yodzis, 1996; Minchella & Scott, 1991; Werner & Peacor, 2003).

Parasites are often neglected components of interaction networks (Lafferty et al., 2008) and ecological communities more generally (Carpenter, Kitchell, & Hodgson, 1985; DeLong et al., 2015; Lafferty, Dobson, & Kuris, 2006). Indeed, we lack a thorough understanding of how parasitism is affected by other types of trophic interactions since parasites feed on or infect species larger than themselves, making them harder to quantify using standard ecological methods (DeLong et al., 2015; Lafferty et al., 2006). For instance, one hypothesized interaction is that predation may decrease parasitism directly by reducing host population density (Anderson & May, 1981; Strauss et al., 2016) or by selectively killing infected hosts (Duffy, Housley, Penczykowski, Cáceres, & Hall, 2011; Packer, Holt, Hudson, Lafferty, & Dobson, 2003). Conversely, predators may indirectly increase rates of parasitism if prey are forced to trade‐off anti‐parasite traits with predator avoidance traits (Rohr et al., 2015). Recent theoretical work has investigated how both density‐ and trait‐mediated indirect effects (e.g., predator effects on specific prey traits, Werner & Peacor, 2003) can alter disease systems (Rapti, Stewart Merrill, Mueller‐Brennan, Kavouras, & Cáceres, 2019), though we still need further empirical work to better understand how predation and parasitism can interact to alter patterns within ecological communities.

It has previously been shown that through nonconsumptive mechanisms, predation risk can influence life‐history traits that mediate parasitism in *Daphnia* (Bertram et al., 2013). Traits such as adult body size, age of reproduction, as well as the number and size of offspring each contribute to the vulnerability of *Daphnia* to both predation and parasitism (Weber & Declerck, 1997). For example, if *Daphnia* increase their size in response to predation to avoid consumption (Duffy et al., 2011), they can become more likely to contact parasites due to increased filtration rates associated with larger body sizes (Hall et al., 2007; Kirk, Luijckx, Stanic, & Krkošek, 2019). Importantly, multiple host traits may shape different stages of the infection process (Hall, Bento, & Ebert, 2017; Stewart Merrill, Hall, Merrill, & Cáceres, 2019)**,** causing nonconsumptive effects to operate differently at different stages of infection. In the current study, we are concerned with two such stages: susceptibility to initial infection, and within‐host replication once infected (Duffy et al., 2011). Predators may therefore affect parasitism by altering host susceptibility or immune capability; reciprocally, within‐host infection intensity may alter host reproduction, shifting the abundance of prey available for predators.

The effects of environmental stressors on reproduction are one of the most distinguishable indirect effects. Parasitic infection has the capacity to influence important traits within *Daphnia*, including increases or decreases of reproductive output in females. For example, Chadwick and Little (2005) showed evidence that *Daphnia* alter their life history in response to parasitic infection to produce more offspring earlier in life. Indeed, these effects may scale‐up to alter food web dynamics, as parasites can have large indirect effects in ecosystems (Buck, 2019). Additionally, complications may arise due to the differing effects of both predator and parasite presence on either male versus female *Daphnia*. Few studies investigate nonconsumptive effects in male *Daphnia*, as asexual reproduction is typical under favorable conditions (Pijanowska & Stolpe, 1996; Schwartz & Hebert, 1987). Therefore, it is unclear how males in isolation will react to the presence of predator kairomones, and whether this will influence their susceptibility to infection.

We used *Daphnia magna*, one of its microsporidian parasites (*Ordospora colligata*), and predator cues from the glassworm *Chaoborus* to investigate nonconsumptive effects in a simplified food web. We sought to investigate how predator cues affect *Daphnia* susceptibility to infection and the subsequent parasite growth within both male and female *Daphnia*. We also investigated if predator cues and parasitic infection jointly influence *Daphnia* reproductive output, which can ultimately feedback to determine the number of new susceptible hosts available to the parasite, as well as available to predators for consumption.

## 2 MATERIALS AND METHODS

### 2.1 Study system

We used the *Daphnia magna–O. colligata* host–parasite system (Ebert, 2005), in which the microsporidian parasite infects the gut epithelium of the freshwater crustacean host. The parasite grows intracellularly before eventually lysing the cell and being shed into the environment. Transmission occurs when foraging *Daphnia* consume infective spores while filter‐feeding. *Daphnia* exhibit phenotypic plasticity, influencing various aspects of parasitic transmission (Stibor & Lüning, 1994; Yin, Laforsch, Lohr, & Wolinska, 2011). Morphological, behavioral, and physiological changes have been previously recorded in response to predator presence (Weiss, 2019). For example, one source of phenotypic plasticity is in response to threats of predation by *Chaoborus* larvae, which are able to consume *Daphnia* in freshwater environments (Ketola & Vuorinen, 1989). Therefore, introduction of *Chaoborus* larvae chemical cues (kairomones) allows for the study of *Daphnia* response without the associated consumptive effects. To control for genetic variation, the experiment was conducted with one Finnish clone (FI‐OER‐3‐3) and one strain of the parasite (OC3) which was originally isolated in this clone.

### 2.2 Experimental design

We tested for the effects of predator cues on *Daphnia* that have been exposed to parasites, and the joint effects of predator cues and parasites on *Daphnia* fecundity. We were primarily interested in the interaction chain of predator → prey → parasite; therefore, we manipulated the presence of predator cues by imposing three treatments on individually housed *Daphnia* that were exposed to the parasite. The no predator cue treatment received no predator cue, the early predator cue (EPC) received predator cues every 3 days for 10 days prior to and during exposure to the parasite, and the sustained predator cue (SPC) received predator cues every 3 days for 10 days prior to, during, and after exposure to the parasite. Each treatment was replicated 96 times. Due to the ability of *Daphnia* to respond within a single generation to predator cues in the environment, we were interested in whether phenotypic changes induced during the juvenile period would be sustained despite the removal of persistent cues in the EPC treatment (Luhring, Vavra, Cressler, & Delong, 2018; Walsh, Cooley, Biles, & Munch, 2015). In natural systems, the duration of predator cues in the environment may be mediated by abiotic factors such as lake size (e.g., large spaces where predators can move toward and away from the prey through time versus small spaces where predator movement is restricted and cues are constant) or biotic factors such as how quickly an insect larvae can develop into an adult and leave the water body (e.g., *Chaoborus*). These treatments allowed us to evaluate changes in responses of the host such as body size, fecundity, parasite infection, and parasite infection intensity in relation to sustained or punctuated exposure to predator cues. We introduced predator cues from buckets containing approximately 30 *Chaoborus* in 2 L of artificial *Daphnia* medium (ADaM; Klüttgen, Dülmer, Engels, & Ratte, 1994).

In order to determine traits that govern *Daphnia* susceptibility to infection, we randomly selected a combined total of 288 uninfected male and female *Daphnia* neonates from cultures and individually transferred them to 80 ml mesocosms filled with ADaM (day 0). The *Daphnia* in the EPC and SPC treatments received 1.5 ml of *Chaoborus*‐conditioned media. After 72 hr, we transferred all individuals to new mesocosms containing ADaM, batch‐cultured algae (*Monoraphidium minutum*), and 1.5 ml of *Chaoborus*‐conditioned media for the EPC and SPC treatments. We repeated this procedure every 3 days for the duration of the experiment; however, only individuals in the SPC treatment group received 1.5 ml doses of *Chaoborus*‐conditioned media after parasite exposure (day 10). We note that transferring individuals into new medium every 3 days limited the likelihood of re‐infection from the environment; therefore, parasite load should be primarily determined by within‐host growth (Kirk et al., 2018).

On day 10, we transferred individuals to new mesocosms and exposed the EPC and SPC treatments to the predator cue. We then created spore doses by homogenizing infected stock *Daphnia* using a mortar and pestle and quantified the concentration of spores in the homogenate using a hemocytometer. We then introduced 1 ml (estimated 5,760 spores) to each mesocosm. All individuals were transferred to new mesocosms after 3 days of parasite exposure.

During transfers, we removed and counted offspring to determine female *Daphnia* fecundity. We measured and dissected individuals that died during the course of the experiment (*n* = 23) in order to quantify the presence and intensity of *O. colligata* infections and confirmed four infections in those that died early. We concluded the experiment on day 32 by conducting a final offspring count and sacrificing all surviving *Daphnia* to assess infections via dissection and phase‐contrast microscopy. Of the 265 *Daphnia* that lived until the conclusion of the experiment, 34 were lost due to dissection failure, leaving a total of 231 *Daphnia* for which we have infection data from the end of the experiment plus the additional four infected individuals that died earlier.

### 2.3 Statistical analysis

We used a generalized linear model (GLM) with a binomial response and logit link to determine if there was an effect of sex (male/female) on the probability of becoming infected (binary response). We then analyzed the effects of the predator cue treatments on infection status separately for the sexes again using GLMs with binomial responses and logit link functions.

We investigated whether sex and predator cue treatments had an effect on infection intensity. We subset the data to include only infected individuals that lived until the end of the experiment (*n* = 231), then used a vector generalized linear model (VGLM; *VGAM* package in R) to test for an effect of sex on infection intensity. The VGLM allowed us to use zero‐truncated negative binomial regression (ZTNBR) since infection intensity is comprised of count data for which values of zero cannot occur. Next, we tested if predator cue treatments affected female infection intensity. We did not test for the effect of predator cue treatments on male infection intensity as the total number of males that were infected across the three predator cue treatments was low (*n* = 12).

We used separate linear models to test for effects of predator cue treatments on female size and fecundity. We chose these life‐history traits because they can potentially influence parasitism in *Daphnia* via the effects of body size on disease transmission (Hall et al., 2007; Kirk et al., 2019) and within‐host parasite intensity (Hall, Simonis, Nisbet, Tessier, & Cáceres, 2009), and linkages between host fecundity and host–parasite outcomes (Hurd, 2001). We used a linear model to predict the number of offspring produced by females across the predator cue treatments (i.e., infected and uninfected females were combined), as well as a linear model with predator cue treatment, infection status, and their interaction as predictors.

Finally, we tested four separate linear models to investigate the effects of infection intensity and predator cue treatment on offspring production in infected females. The predictors included in these models were as follows: (a) only infection intensity; (b) only predator cue treatment; (c) both predictors but no interaction; or (d) both predictors and their interaction. We compared the models using the Akaike information criterion (AIC).

## 3 RESULTS

**FIGURE 1:**
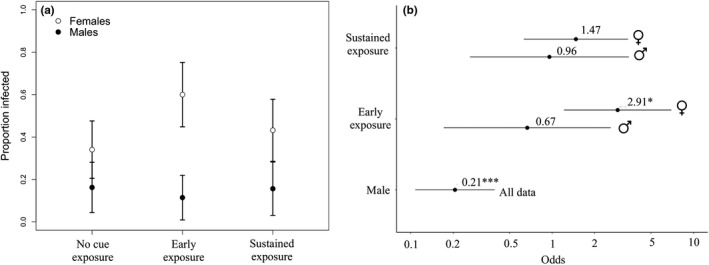
(a) Proportion of females and males that became infected in the no predator cue, early exposure, and sustained exposure treatments. Error bars represent 95% confidence intervals determined from the standard error of the proportions. (b) The effects of predator cue treatments and sex on the likelihood of becoming infected. Effects of predator cues were estimated separately for males and females (illustrated by the separate male and female symbols). Data from all predator cue treatments were pooled for determining the effect of sex. Odds for the effects of sex are related in terms of the effect of being male on becoming infected. Males were significantly less likely to become infected, and early exposure to predator cues significantly increased the likelihood of females becoming infected. Error bars represent 95% confidence intervals

**FIGURE 2:**
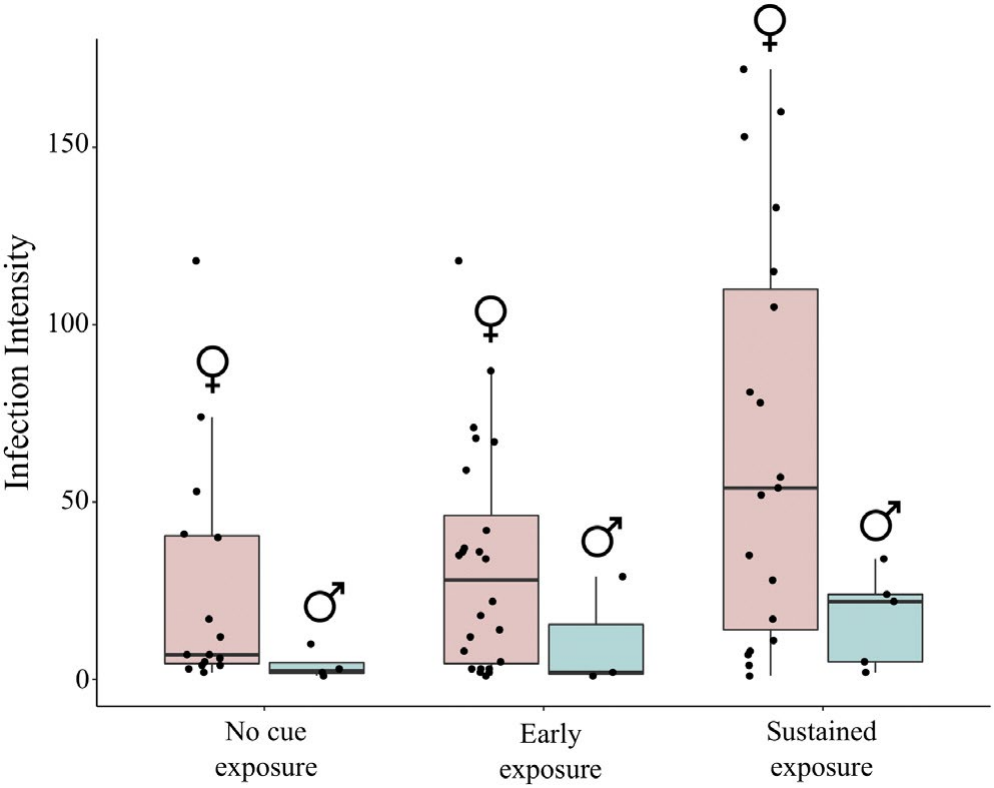
Infection intensity in infected females and males across predator cue treatments. Only infected individuals (i.e., those with nonzero infection loads) are shown. Zero‐truncated negative binomial regression (ZTNBR) revealed that females had significantly greater parasite intensities than males (male estimate −1.46 ± 0.429 *SE*; *p* < .001; *n* = 70). Infected females (*n* = 58 total) that were exposed to predator cues throughout the experiment (*n* = 19) had significantly higher parasite intensities than the females that were not exposed to the predator cue (0.983 ± 0.423 *SE*; *p* = .020), though parasite intensity was not significantly affected by early exposure (*p* > .5). These qualitative results were all consistent when including uninfected *Daphnia* (*n* = 231 total *Daphnia*, *n* = 130 females, *n* = 100 males) and fitting with regular negative binomial regressions rather than ZTNBR

We analyzed infection status in 235 *Daphnia* (131 females and 104 males), 231 of which lived until the end of the experiment. Females were ~5× more likely than males to become infected (*p* < .0001; Figures 1 and 2). Early exposure to predation risk increased the likelihood of being infected ~threefold in females (*p* = .017; Figures 1 and 2), demonstrating a nonconsumptive effect of the predator on the host–parasite interaction, while sustained exposure had a smaller positive but nonsignificant effect (*p* = .37, Figures 1 and 2). Conversely, we were unable to detect any effects of predator cues on infection rate in males (both *p* > .5; Figures 1 and 2).

Analyses revealed that females had significantly greater parasite intensities than males (male estimate −1.46 ± 0.429 *SE*; *p* < .001; *n* = 70, Figure 2). Additionally, infected females (*n* = 58 total) that were exposed to predator cues throughout the experiment (*n* = 19) had significantly higher parasite intensities than the females that were not exposed to the predator cue (0.983 ± 0.423 *SE*; *p* = .020), though parasite intensity was not significantly affected by early exposure (*p* > .5). These qualitative results were all consistent when including uninfected *Daphnia* (*n* = 231 total *Daphnia*, *n* = 130 females, *n* = 100 males) and fitting with regular negative binomial regressions rather than ZTNBR. Exposure to predator cues early and in a sustained manner did not significantly affect female body size.

**FIGURE 3:**
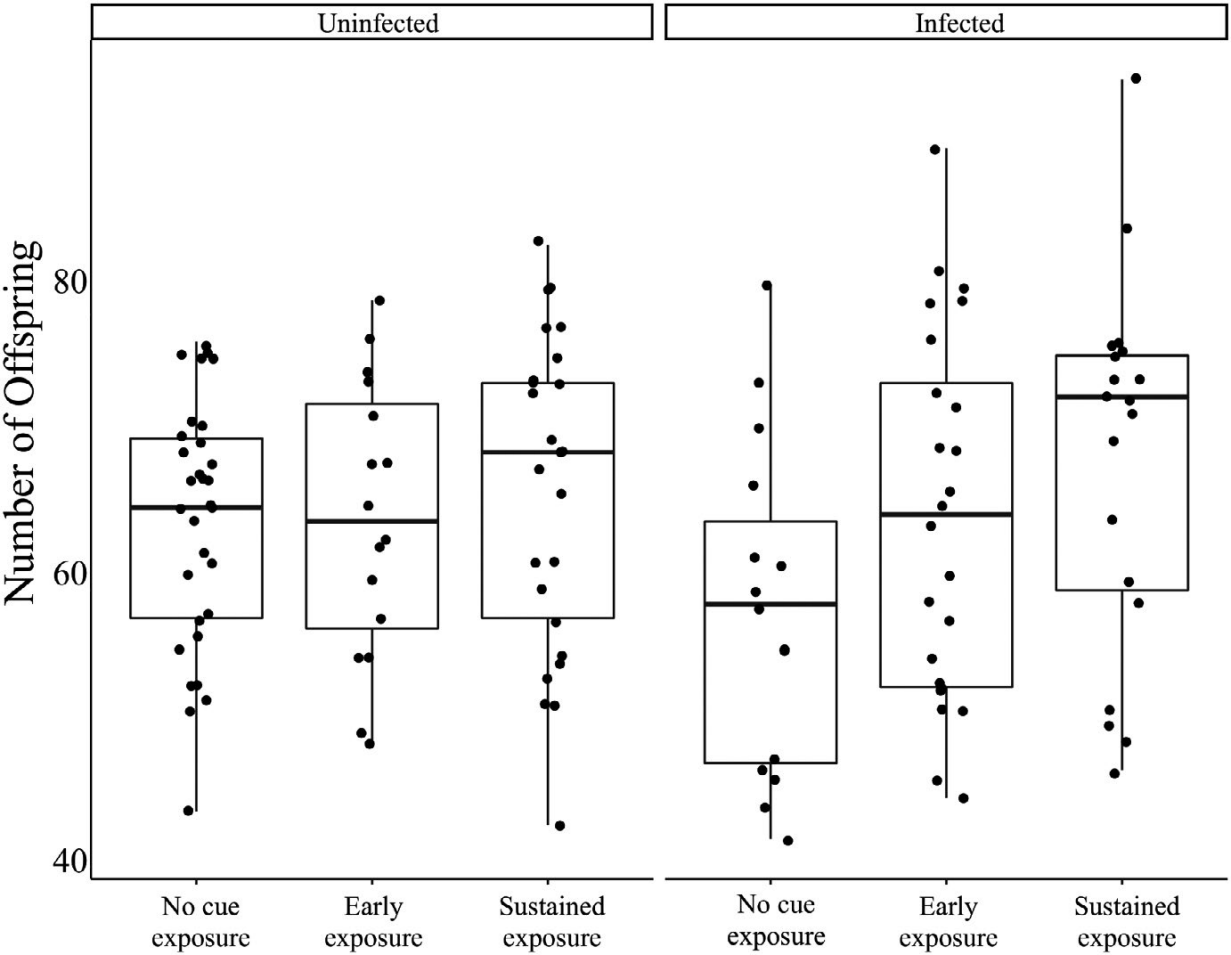
Offspring production across predator cue treatments, including both infected and uninfected individuals. Including both infected and uninfected females, we found that females in the sustained exposure treatment had significantly more offspring than the no predator cue treatment (5.29 ± 2.42 *SE*; *p* = .0304), a pattern that was not detected in the early exposure treatment (*p* = .35). Our linear model that included an interaction between predator cue treatment and infection status did not detect any significant predictors of number of offspring, though being infected had a nearly significant and negative effect (−6.368 ± 3.600 *SE*; *p* = .079), while being infected in the sustained predator cue treatment had a nearly significant and positive effect (8.364 ± 5.008; *p* = .097), suggesting that being infected in the no predator cue treatment resulted in low offspring production

Including both infected and uninfected females, we found that females in the sustained exposure treatment had significantly more offspring than the no predator cue treatment (5.29 ± 2.42 *SE*; *p* = .0304), a pattern that was not detected in the early exposure treatment (*p* = .35; Figure 3). Our linear model that included an interaction between predator cue treatment and infection status did not detect any significant predictors of offspring number, though microsporidian infection had a nearly significant and negative effect (−6.368 ± 3.600 *SE*; *p* = .079), while infection in the sustained predator cue treatment had a nearly significant and positive effect (8.364 ± 5.008; *p* = .097), suggesting that microsporidian infection in the absence of predator cues resulted in low offspring production (Figure 3).

**FIGURE 4:**
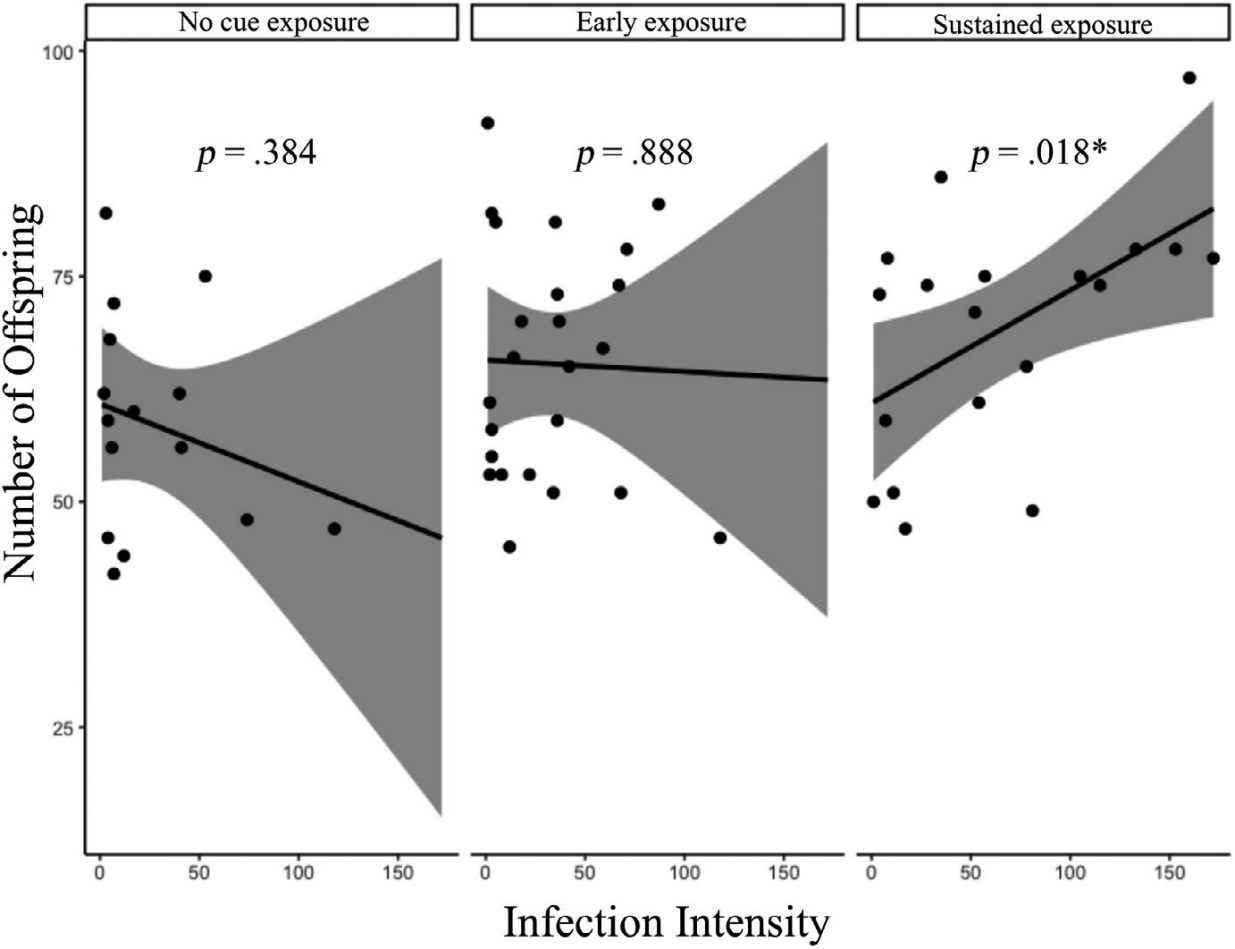
Number of offspring produced by infected females versus their infection intensity. Lines represent linear regressions for offspring produced as a function of infection intensity for infected females, calculated within each predator cue treatment, and shaded regions represent 95% confidence intervals. There was no significant relationship in the no predator cue treatment (*p* = .384) or early exposure treatment (*p* = .888), but a significant positive effect of infection intensity on number of offspring in the sustained exposure treatment (*p* = .018)

None of the four linear models investigating offspring production in infected females performed significantly better than the others (within Δ2 AIC); therefore, we report the results for each here. The linear model that only included infection intensity as a predictor identifies a significant positive effect of infection intensity on offspring production in infected females (0.080 ± 0.038; *p* = .0405). The predator cue treatment model found a significant positive effect of the sustained treatment (10.72 ± 4.49; *p* = .0204) but not the early treatment (6.69 ± 4.28; *p* = .123) on offspring production. The model with both predator cue treatment and infection intensity did not identify any predictors as significant, although sustained treatment was positive and nearly significant (8.31 ± 4.75; *p* = .0855). The model that included all predictors and their interaction only identified the infection intensity by sustained treatment interaction as nearly significant (0.212 ± 0.112; *p* = .0651). We also ran separate linear regressions for offspring production regressed against female infection intensity across the three predator cue treatments and found a significant positive effect in the sustained treatment (0.125 ± 0.0478; *p* = .0178; Figure 4), but did not find an effect in the no predator cue (*p* = .384) or early cue treatments (*p* = .888). Finally, our linear model that used predator cue treatment as a predictor of offspring production in uninfected females did not identify early or sustained predator cues as significant.

## 4 DISCUSSION

Predation risk can influence patterns of prey fecundity and can indirectly affect parasitism (Declerck & Weber, 2003; Tollrian, 1995; Yin et al., 2011). Here, we demonstrate nonconsumptive effects of predators that mediate prey susceptibility to infection and their subsequent ability to resist within‐host parasite growth. Our results demonstrate that indirect interactions occur between predators and parasites, but that the strength of such effects is complex and can depend on both how long the host is exposed to the cues as well as host sex.

Predator cues affected the likelihood of female *Daphnia* becoming infected (Figures 1 and 2), demonstrating a predator–host–parasite indirect interaction. Early exposure significantly increased the likelihood of a female host becoming infected, while sustained exposure had a smaller positive but nonsignificant effect (Figures 1 and 2). These results can be explained by either the predator cues increasing female *Daphnia* filtration rate (thereby increasing contact rate with the environmentally transmitted parasite) or by increasing the probability of infection after contact (Kirk et al., 2019). If the former is responsible for our observations, we speculate that *Daphnia* increase foraging rates to acquire resources in the face of predation (potentially for increased reproduction) (Figure 4). Alternatively, if this result is due to an increased probability of infection after contact, it may be due to an impaired immune response as a result of predator exposure (perhaps also due to a trade‐off associated with increased reproduction) (Gipson & Hall, 2018). This would be in contrast to Bertram et al.'s (2013) finding in the *Daphnia dentifera*–*Metschnikowia* host–parasite system, where reproductive output decreased in response to fish kairomones and was unaffected by *Chaoborus* cues. Interestingly, Bertram et al. (2013) also found no effect of *Chaoborus* cues on body size and a positive effect of the cues on infection risk, suggesting that our results shown here (Figure 1) are not restricted to this *Daphnia*–parasite system. In addition to investigating these questions in a new system, we build upon this previous work by also examining if the duration of predator cue exposure affects the host–parasite interaction for both infection rates and subsequent within‐host dynamics.

Here, we found significant evidence for infection risk increasing when hosts were exposed to predator cues early in life but not when they were exposed throughout their life in the sustained treatment. This is a nonintuitive finding, as there were no methodological differences between the early exposure and sustained exposure treatments during the period preceding or during when hosts were exposed to the parasite and could become infected. This suggests that any differences between the treatment results could be due to processes occurring after hosts have already become infected. Speculatively, if *Daphnia* exhibit a general defensive response to enemies that enhances their immune system when exposed to predator cues over long periods of time, sustained exposure to predators could help some *Daphnia* individuals clear infections after they become infected. Different types of defenses may be upregulated at different times, whereby different responses would be associated with the ability to become infected and infection intensity once infected. However, this is not supported by our separate finding that sustained exposure increased infection intensity (Figure 2) as higher infection intensities should make it more difficult for hosts to clear infection, though we note that infection intensity is highly variable (Figure 2), which occurs due to the stochastic nature of parasite growth within the host (Kirk et al., 2018). In sum, although we are unable to explain why we did not find a significant effect of sustained exposure on becoming infected, our results of early exposure are clear evidence that link predators to infection rates via nonconsumptive effects.

Predator cues increased parasite infection intensity and would therefore also increase costs of infection to hosts via decreased survival in natural settings (Kirk et al., 2018). We found that individuals chronically exposed to predator cues had the greatest infection intensities, followed by those that were exposed early, with *Daphnia* that were unexposed to cues supporting the fewest parasites (Figure 2). Our results show that predator cues not only have an effect on host susceptibility to infection, but also on infection progression within the host. Notably, these different stages of infection mediated by nonconsumptive effects likely have distinct genetic architectures (Hall et al., 2017), meaning the traits associated with host susceptibility and resistance to within‐host growth may evolve separately. Additionally, our results highlight the large amount of intraspecific variation in infection load (Figure 2) and fecundity (Figure 3), despite the fact that the experiment was conducted with only one host clone and one parasite isolate. Both of these results confirm previously found high‐levels of intraspecific variation in this system (Kirk et al., 2018), and we speculate that the wide range of infection intensity arises from the stochastic nature of the within‐host dynamics. Similar variation in other systems may make it more difficult to detect the effects of predators and predator cues on host–parasite interactions.

Our results suggest that key life‐history traits may characterize the trade‐off between predation and parasitism. Indeed, previous studies have shown that, depending on the type and size of the predator, organisms may respond to predator cues by increasing reproduction (Gleichsner, Cleveland, & Minchella, 2016; Vale & Little, 2012). One possible explanation for the observed positive relationship between infection intensity and offspring production in infected females is that *Daphnia* may be able to sense infection intensity (and the corresponding survival costs) and increase their reproductive rate (at least over the short term). Alternatively, *Daphnia* may respond to predator cues by increasing reproductive output, but this additional expenditure of energy and resources leads to a decreased ability to fight infection, resulting in an increased parasite intensity. Since we only found a significant relationship between infection intensity and offspring production in the sustained predator cue treatment (Figure 4), we speculate that the latter is more likely, as there was no clear relationship in the no predator cue or early cue treatments. Moreover, infected females in the no predator cue treatment tended to have lower (though nonsignificant) offspring production (Figure 3). We also note that the increase in reproductive output independent of body size provides evidence of a switch in life history strategy in response to perceived environmental threats. Future studies testing which cues *Daphnia* are responding to first may be able to resolve the direction of causation and provide deeper insights into the mechanisms underlying these interactions.

Beyond simply demonstrating indirect effects between predators and parasites, our work demonstrates that nonconsumptive effects may be sex‐specific, necessitating an understanding of how individual differences affect indirect interactions. Males were less likely to become infected and supported fewer parasites relative to females (Figures 1 and 2). We note that lower infection intensity in males is likely predominantly a result of their smaller body size relative to females, as a model using only body size reveals it a significant predictor of infection intensity (1.17 ± 0.363; *p* = .001), and this model is not significantly better than a model using only sex to predict infection intensity (ΔAIC = 0.379). However, differences in body size do not explain why males were ~5× less likely than females to become infected, as male and female neonates were not disinguishable by size at the start of the expermiment when individuals were exposed to the parasite. Males are often thought to have lower immune functioning and greater parasite intensities (Zuk & McKean, 1996), although the opposite may occur in this system because of particularly high female investment in reproduction or because the relatively small males have fewer available cells for the parasites to infect (Duneau, Luijckx, Ruder, & Ebert, 2012). More subtly, we found that female but not male infection risk was affected by predator cues (Figures 1 and 2). Past work (Gipson & Hall, 2018; Zuk & McKean, 1996) has shown that the trade‐off between defense (including immunity) and reproduction is fundamentally different for males and females, and our results suggest that this may be the case in this system as well. Importantly, however, any potential effects of predator cues in males in this system will be harder to identify due to lower male infection rates, signifying that males may also experience nonconsumptive effects but that they will be more difficult to detect. More generally, predator‐induced nonconsumptive effects in types of individuals (in this system males) that have low infection rates and low parasite intensities will likely be harder to detect statistically, but will also be less impactful at the population level due the low force of infection of these individuals.

The indirect effects of predators on prey life history and parasitism at the individual level are likely to translate to changes in population‐level disease dynamics. Indeed, reproduction‐immunity trade‐offs can have consequences for patterns of infection, the strength of nonconsumptive effects, and likely for broader patterns of disease spread and host population dynamics (Duneau & Ebert, 2012). Predator cues caused an increase in both *Daphnia* susceptibility to infection and within‐host parasite growth, meaning that parasites should be more common in the environment when predators are abundant. As a result, we may expect greater parasite prevalence when predation risk is high, suggesting facilitation is occurring between the predator and parasite. However, predators may also affect host–parasite dynamics by changing host abundances via predation (i.e., density‐mediated indirect interactions). The overall effects of predators on disease in the prey population will then depend on the relative importance of predation and nonconsumptive effects.

Our study characterizes indirect interactions between predators and parasites that operate across stages of host–parasite interactions and suggests that trade‐offs exist between defending against one or the other enemy. Such trade‐offs are likely mediated by differential investment in key life‐history traits, as well as potential differences between male and female *Daphnia*. Predators, prey, and their parasites can form complex (and common) (Lafferty et al., 2008) subnetworks in food webs, requiring a nuanced understanding of how indirect effects link life‐history traits, trophic interactions, and community structure and function.

## CONFLICT OF INTEREST

None declared.

## AUTHOR CONTRIBUTIONS

**Nicolette Zukowski:** Conceptualization (equal); Investigation (equal); Methodology (equal); Writing‐original draft (lead); Writing‐review & editing (equal). **Devin Kirk:** Conceptualization (equal); Formal analysis (lead); Investigation (equal); Methodology (equal); Supervision (equal); Visualization (lead); Writing‐review & editing (equal). **Kiran Wadhawan:** Conceptualization (equal); Investigation (equal); Methodology (equal); Writing‐review & editing (equal). **Dylan Shea:** Conceptualization (equal); Investigation (equal); Methodology (equal); Writing‐review & editing (equal). **Denon Start:** Conceptualization (equal); Investigation (equal); Methodology (equal); Writing‐review & editing (equal). **Martin Krkošek:** Conceptualization (equal); Funding acquisition (lead); Investigation (equal); Methodology (equal); Resources (lead); Supervision (equal); Writing‐review & editing (equal).

## Data Availability

Data used to generate the results and figures are available from the Dryad Digital Repository: https://doi.org/10.6078/D1597
